# Atypical bromethalin intoxication in a dog: pathologic features and identification of an isomeric breakdown product

**DOI:** 10.1186/s12917-015-0554-y

**Published:** 2015-09-28

**Authors:** Maria C. Bates, Patrick Roady, Andreas F. Lehner, John P. Buchweitz, B. Heggem-Perry, Stephane Lezmi

**Affiliations:** College of Veterinary Medicine, Department of Pathobiology & Veterinary Diagnostic Laboratory, University of Illinois, 2001 S. Lincoln Ave, Urbana, IL 61802 USA; Diagnostic Center for Population and Animal Health, Toxicology Section, Michigan State University, 4125 Beaumont Rd, Lansing, MI 48910 USA; Department of Pathobiology and Diagnostic Investigation, College of Veterinary Medicine, Michigan State University, 784 Wilson Rd, East Lansing, MI 48824 USA; College of Veterinary Medicine, University of Illinois, Veterinary Teaching Hospital 1008 W. Hazelwood Dr., Urbana, IL 61802 USA

## Abstract

**Background:**

Definitive post mortem confirmation of intoxication by the neurotoxic rodenticide bromethalin can be challenging. Brain lesions are not specific and detection of bromethalin and its metabolites are unpredictable due to rapid photodegradation and inconsistent behavior in tissues.

**Case presentation:**

A 2-year-old dog presented with rapid onset of severe muscle tremors and death within hours after a known ingestion of a reportedly low dosage of bromethalin and subsequent decontamination using activated charcoal. Marked meningeal hemorrhages and multifocal myelin sheath vacuolation were observed in the brain. A marked reactive astrocytosis and neuronal hypoxia/necrosis were identified using immunohistochemistry (IHC) for glial fibrillary acidic protein (GFAP) and for neuron specific protein (NeuN). Bromethalin exposure and tissue absorption was confirmed by identification of one of two isomeric 543.7 molecular weight (MW) breakdown products in the patient’s adipose and kidney samples using gas chromatography (GC) combined with tandem quadrupole mass spectrometry (MS/MS).

**Conclusions:**

The severity of clinical signs and subsequent death of this dog was not expected with the low dosage of bromethalin reportedly ingested, and the use of activated charcoal possibly precipitated a hypernatremic status. Meningeal hemorrhages are atypical of bromethalin intoxication, and might have been caused by hyperthermia, secondary to tremors or hypernatremia. Identification of one of two isomeric breakdown products in the adipose tissue and kidney provides an additional molecule to the toxicologic testing regime for bromethalin intoxication.

## Background

Bromethalin is a potent neurotoxic rodenticide, developed in the late 1970’s to combat rodent resistance to anticoagulant rodenticides [1]. Ingestion of rodent baits by companion animals is a common toxicologic emergency, and may become more frequently observed with a recent ban of second generation anticoagulant rodenticides, limiting the options for effective products available to consumers [1, 2]. There is no specific antidote for bromethalin intoxication and clinical signs in dogs are typically dependent on the dose involved. Large doses (2.38-5.6 mg/kg, LD50: 4.7 mg/kg in the dog) lead to an acute onset of clinical signs (within 2 to 12 hours) including severe muscle tremors, seizures, hyperesthesia, hyperexcitability and death due to respiratory paralysis [3]. Lower doses tend to exhibit a prolonged time course with clinical signs developing 2–4 days after ingestion. These signs may mimic those seen with spinal cord compression secondary to disc disease (ataxia progressing to decreased proprioception, loss of deep pain response, upper motor neuron urinary signs), and may progress over 1–2 weeks to include muscle tremors, hyperexcitability, rigidity, seizures, coma and death [3]. The lowest published dose leading to clinical signs and death in a dog is 0.45 mg/kg [2]. Interestingly, similar clinical signs have also been described in a human intoxicated with low dose of bromethalin [4].

Treatment is typically focused on decontamination (including administration of activated charcoal), monitoring for the development of clinical signs, and providing supportive care. Paradoxically, in our case the administration of activated charcoal could have precipitated the disease by inducing hypernatremia.

Confirmation of bromethalin intoxication can be challenging as bromethalin is rapidly photodegraded [3]. Because tissues samples collected at necropsy are not typically protected from light, and toxicologic analyses are not performed in dark rooms, as well as for other unknown causes, tissue residues are often of too low a concentration to be detected by conventional methods, i.e. Selected Ion Monitoring (SIM) or full scan data acquisition from single quadrupole gas chromatography/mass spectrometry (GC/MS). Tandem quadrupole mass spectrometry offers a highly sensitive means of detecting bromethalin breakdown products due to its significant reduction of background interference by the second quadrupole as well as the enhanced specificity offered by the breakdown products’ exceptionally high mass/charge (m/z) ratios. It likely offers several orders of magnitude greater sensitivity than SIM on single quadrupole GC/MS and was therefore deemed an appropriate detector following gas chromatography (GC).

## Case Presentation

A 5.9 kg, 2- year-old, female spayed, mixed breed dog was submitted to the Veterinary Teaching Hospital emergency service for ingestion of 1 to 1.5 bricks containing 0.01 % bromethalin mouse bait (Tomcat brand, Motomco, Madison WI) between 3–7 hours earlier that day. The patient was clinically normal on presentation and clinical chemistry parameters, including electrolytes, were within normal limits. Per consult with animal poison control, the estimated dose of bromethalin was determined to be low (0.8 mg/kg) and decontamination with close monitoring for development of clinical signs was deemed appropriate therapy. Emesis was induced and a small amount of mouse bait was produced. A 20 % intravenous fat emulsion (Intralipid® manufactured by Fresenius Kabi, Uppsala, Sweden for Baxter Healthcare Corporation, Clintec Nutrition Division, Deerfield, IL 60015 USA) was administered as an 1.5 ml/kg bolus given IV over 15 minutes then 0.5 ml/kg/min for 30 minutes as a constant rate infusion. Activated charcoal (130 mls of ToxiBan® Lloyd, Inc, Shenandoah, IA 51601, USA) containing 104 mg of activated charcoal/ml and 62.5 mg kaolin/ml, with an unspecified concentration of preservatives, suspending agents and water was administered orally. The patient was discharged.

This was a case presentation regarding theemergent veterinary treatment of a poisoned canine with subsequent post-mortem analysis of tissues. Allwork was performed under the consent of the animal’s owner and was not a part of a funded research study. No experimental research was performed that would require the approval of the University of Illinois ethicscommitte.

## Results

### Case outcome

After returning home, the patient’s owners noticed the development of slight ataxia. As instructed, they returned to the emergency room, 2 hours after the initial presentation. The patient re-presented with severe muscle tremors, rigidity, and nystagmus. The patient was administered 1 mg/kg of diazepam rectally and then 0.5 mg/kg intravenously (Hospira Inc, Lake Forest, IL 60045 USA). During these stabilization efforts she became acutely cyanotic and rigid. Cardiac auscultation revealed bradycardia with a faint heart beat and pulse deficits. She was intubated and atropine 0.04 mg/kg (Butler Schein Animal Health, Dublin, OH 43017, USA), and two doses of epinephrine 0.01 mg/kg (IMS Limited, South El Monte, CA 91733, USA) were administered intravenously, though she failed to respond and was found to be in asystole. During CPR and for at least 45 minutes after death, the animal remained in rigid extension. A venous blood sample, collected minutes before death, revealed sodium concentration of 168 mmol/L (normal values 144–151 mmol/L).

### Post mortem findings, histopathology and IHC analysis

**Fig. 1:**
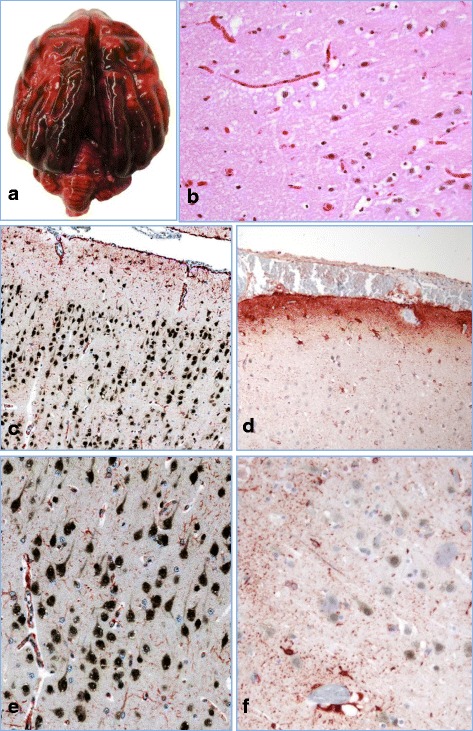
Brain, cortical lesions. **a** Gross image of brain at necropsy showing marked meningeal congestion and hemorrhages. **b** In the cortical grey matter are shrunken and hypereosinophilic neurons as well as mild vacuoles of the neuropil (spongiosis), H&E stain 400x. **c** and **e**) Control canine brain IHC for GFAP (red) and NeuN (black). Note intense black stain of normal neurons and scattered red staining of normal, resting astrocytes (100x and 400x). **d** and **f**): IHC for GFAP and NeuN on brain sections from the bromethalin intoxicated dog: Note marked decrease in NeuN (black) staining in neurons compared to control and increase in GFAP (red) indicating enlargement of astrocyte cell bodies and astrocytic processes in the glia limitans. (100x and 400x)

At necropsy the stomach and entire intestinal tract were filled with activated charcoal. The meninges were diffusely red to dark red, wet and gelatinous (hemorrhagic) (Fig. 1a). Histologically, neurons within the cortical grey matter were occasionally, multifocally slightly shrunken and hypereosinophilic suggesting hypoxia and necrosis (Fig. 1b). The surrounding neuropil was pale and multifocally vacuolated (spongiosis). Cerebral and meningeal blood vessels were severely congested. The leptomeninges were diffusely and markedly expanded by hemorrhage (Fig. 1d).

Histological sections (5 μm) were dewaxed, rehydrated in water and then used for standard immunohistochemical analyses as previously described [5, 6]. Briefly, each section was incubated with one of the primary antibodies (GFAP Dako Z0334 1/500; NeuN Abcam ab177487 1/1000). An adequate secondary biotinylated antibody (Jackson Laboratory, Bar Harbor, Maine) and avidin–biotin–peroxidase complex system (ABC Vector Laboratories, Burlingame, CA, USA) were used to detect primary antibodies. DAB-NiCl (Vector, black deposits) and AEC (Vector, red deposits) were used as chromogens. A slight counter-staining was done using aqueous hematoxylin. Nonspecific binding was controlled by omitting the primary antibody, and using other primary antibodies as isotypic controls.

IHC revealed marked increase in reactivity of astrocytes for GFAP, compared to controls with enlargement of astrocyte soma, and increased astrocytic processes (astrocytosis) perivascularly and within the glia limitans (Fig. 1c-f). Neurons within the cortical grey matter exhibited moderate to marked decreased reactivity for NeuN compared to controls (Fig. 1c-f).

**Fig. 2:**
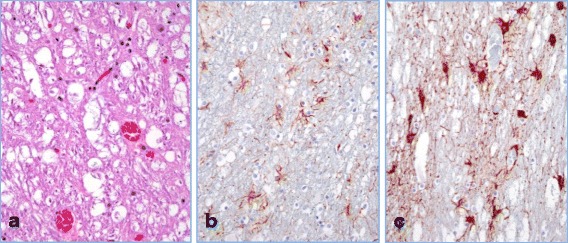
Brain (internal capsule) from dog after bromethalin ingestion and activated charcoal administration. **a** H&E stained section of the intoxicated dog showing vacuolization of myelin sheaths (400x). **b** Control brain white matter stained for GFAP (red) (400x). **c** White matter of canine brain after bromethalin ingestion IHC for GFAP (red), note increased astrocyte cell bodies and astrocytic processes (400x)

Within white matter tracts of the cerebrum, internal capsule, cerebellum and brainstem, the myelin sheaths were multifocally swollen with variably sized, clear vacuoles that occasionally contain scant amounts of finely fibrillar, eosinophilic material (fragmented myelin) (Fig. 2a). Compare to a control brain sample (Fig. 2b), astrocytes within the adjacent cortical gray matter were frequently swollen, and exhibited astrocytosis as previously described (Fig. 2c).

### Toxicologic analyses

To confirm absorption of bromethalin, tissue samples including adipose, liver, kidney, and brain were submitted for toxicologic evaluation. All tissues were initially screened for bromethalin and desmethylbromethalin by simple and less sensitive gas chromatography mass spectrometry (GC-MS) and SIM or Full Scan methodologies. The instrument was an Agilent 7890–5975 GC/MS (Santa Clara, CA) capable of positive mode electron-impact ionization. All samples were negative based on this initial screen. Subsequently, a recently developed gas chromatography tandem quadrupole mass spectrometric (GC-MS/MS) Multiple Reaction Monitoring (MRM) method was developed with an Agilent 7890–7000 GC-MS/MS capable of positive mode electron-impact ionization and equipped with an Agilent Multi-Mode Inlet. This method is sensitive for bromethalin breakdown products and provides additional surrogate markers to screen for bromethalin exposure. In short, the instrumentation enabled generation of MRM settings for bromethalin breakdown products of molecular weights 231, 311, 327, 453, 517, 532, 543 and 547 whose spectra and additional information will be described in a forthcoming article on bromethalin analysis. The most significant and recurring products were two 543.7 MW compounds to either side of the bromethalin chromatographic peak. Each compound represents loss of a methyl group and a molecule of hydrogen fluoride, and one of these structures has been previously described as arising via chemical ionization mass spectrometry (Pasquale-Styles, et al., 2006). MRM settings for these 543.7 MW products were derived by measuring product ions from principal peaks of the compound and then optimizing collision energies for each resulting transition. MRM settings are listed in Table 1. The 543.7 MW breakdown products are of interest in that desmethylbromethalin standard can generate one of the breakdown products, whereas bromethalin standard generates both of them. See Table 2 for the breakdown seen from compound standards. This implies that one of the products can arise from a demethylated intermediate, whereas it is likely that the second one requires the participation of the bromethalin methyl group. It remains to be determined whether the compounds exist as such in sample extracts or in fact are gas chromatographic or even electron impact derived side products from other intermediates, such as from desmethylbromethalin. The operational limits of detection for both bromethalin and desmethylbromethalin were 10 ppm in tissue samples.

**Table 1:** Multiple reaction monitoring (MRM) settings on the GC/MS/MS for bromethalin, desmethylbromethalin and bromethalin’s 543.7 MW breakdown products

Origin	Precursor, m/z	Product, m/z	Dwell, msec	Collision energy, eV
Bromethalin 543.7 mw breakdown product	543	512.5	10	15
Bromethalin 543.7 mw breakdown product	543	405.5	10	30
Bromethalin 543.7 mw breakdown product	399	239	10	35
Bromethalin	577	468	10	10
Bromethalin	468	448	10	10
Bromethalin	468	405	10	30
Bromethalin	226	199	10	25
Desmethyl Bromethalin	563	69	10	20
Desmethyl Bromethalin	313	232	10	5
Desmethyl Bromethalin	311	182	10	35
Desmethyl Bromethalin	231	204	10	20

**Table 2:** Principal breakdown products arising from desmethylbromethalin and bromethalin and their relative amounts based on total ion chromatograph area in standards run at 100 and 1000 ppm. RRT = relative retention time, relative to that of bromethalin

Compound		Desmethyl-bromethalin	DHFDMB-I	Bromethalin	DHFDMB-II
	RRT	0.83	0.85	1.00	1.02
Bromethalin			8.2 %	91.2 %	0.6 %
	Std dev		+/- 1.7 %	+/- 1.7 %	+/- 0.03 %
Desmethylbromethalin		98.8 %	1.2 %		
	Std dev	+/- 0.7 %	+/- 0.7 %		

The GC-MS/MS method made use of tissue extracts prepared in accordance with a previously described method [7]. Briefly, 10 g of sample was mixed with 5 mL 1 N sodium hydroxide and allowed to sit for 5 min then extracted with 100 mL methylene chloride in a 250 mL separatory funnel. 10 mL of 0.1 N sulfuric acid was added to the methylene chloride eluate in a stoppered graduate cylinder and mixed thoroughly. The methylene chloride organic phase was passed through a funnel containing sodium sulfate to remove any water. The organic phase was reduced to 1 mL under a constant stream of nitrogen. The extract was resuspended to a final volume of 4 mL with 1:1 methylene chloride:cyclohexane and purified by gel permeation chromatography (GPC). The fraction eluting from 130 to 200 mL was reduced to 3 mL by rotary evaporator then taken to dryness under a constant stream of nitrogen. The sample was resuspended in 100 uL methanol in a small vial to be analyzed by gas chromatography tandem quadrupole mass spectrometry (GC-MS/MS).

**Fig. 3:**
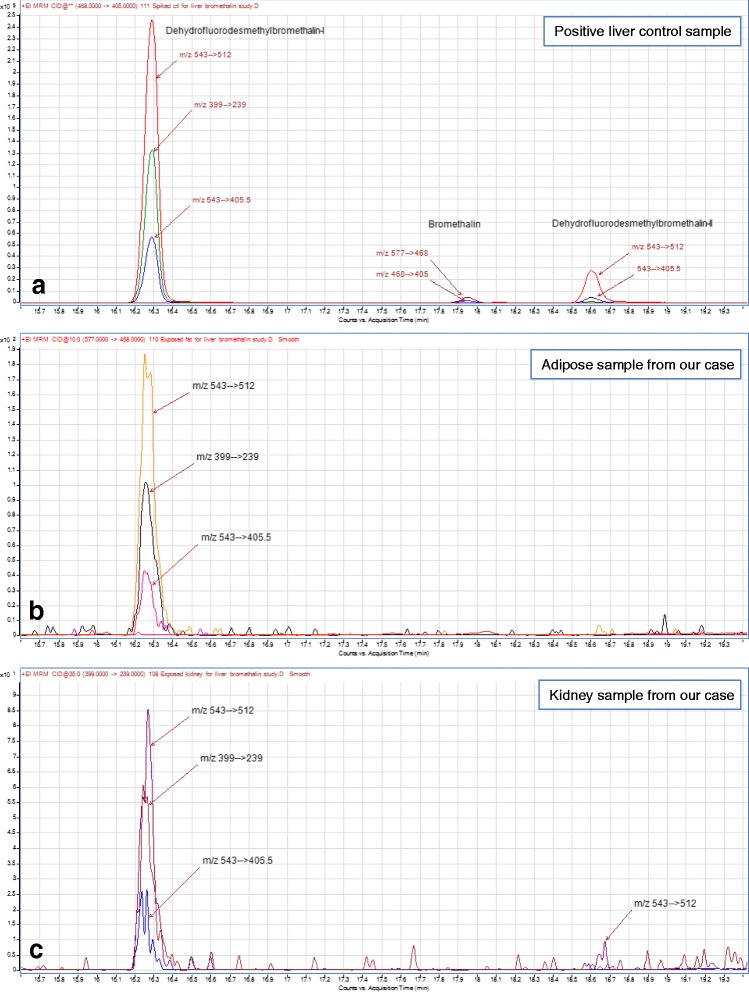
Application of GC-MS/MS bromethalin MRM method. **a** Bromethalin spiked at 100 ppm into control liver sample. Note bromethalin peak at 17.95-min RT, and principal 543.7 MW breakdown peaks (detected with sensitive m/z 543-derived MRMs) at 16.3-min and 18.6-min retention times, respectively. **b** Fat sample collected from our dog. Note that only the 16.3-min bromethalin breakdown compound was present. **c** Kidney from the same dog shows the 16.3-min peak and a trace of the 18.6-min peak

Bromethalin analytical standard was generously supplied by the US Environmental Protection Agency (US EPA) National Pesticide Standard Repository. Desmethylbromethalin standard was from Toronto Research Chemicals (Toronto, Ontario, Canada). IUPAC chemical names were obtained by use of ChemDraw Professional software version 15.0.0.106 (Perkin Elmer, Waltham, MA). Analytical screening of bromethalin, desmethylbromethalin, and physicochemical breakdown products in adipose fat, brain, kidney, liver, and stomach content was performed by GC-MS/MS using two 15 m × 0.250 mm × 0.25 um DB5-MS capillary columns^i^ joined with a “T” configuration to enable post-run backflushing. The GC oven temperature was held at 15 °C for 3 minutes and then ramped to 260 °C at a rate of 20 °C/minute The oven temperature was maintained for 10 minutes then increased at 60 °C/minute to 320 °C (held 8-minutes) for a total run time of 27.5 minutes. Helium was used as the carrier gas with an inlet pressure of 18.1 psi. The inlet configuration was set to pulsed splitless mode and 5 uL of sample was injected for analysis. The inlet temperature was held at 130 °C for 1 min. and then ramped to 325 °C at a rate of 600 °C/min for 5 min. The transfer line temperature was set to 280 °C. The collision cell used helium as the quench gas at 2.25 mL/min, nitrogen as the collision gas at 1.5 mL/min, and a collision energy ranging from 5 to 35 volts depending on analyte. Results for kidney and adipose samples as well as a spiked liver control are shown in Fig. 3a. The positive control for this study consisted of unexposed bovine liver spiked with bromethalin in methanolic solution to 100 ppm. The negative control was the same liver matrix treated with solvent only, and its examination produced a flat baseline in comparison to the chromatographic peaks seen in the spike or samples (result not shown).

**Fig. 4:**
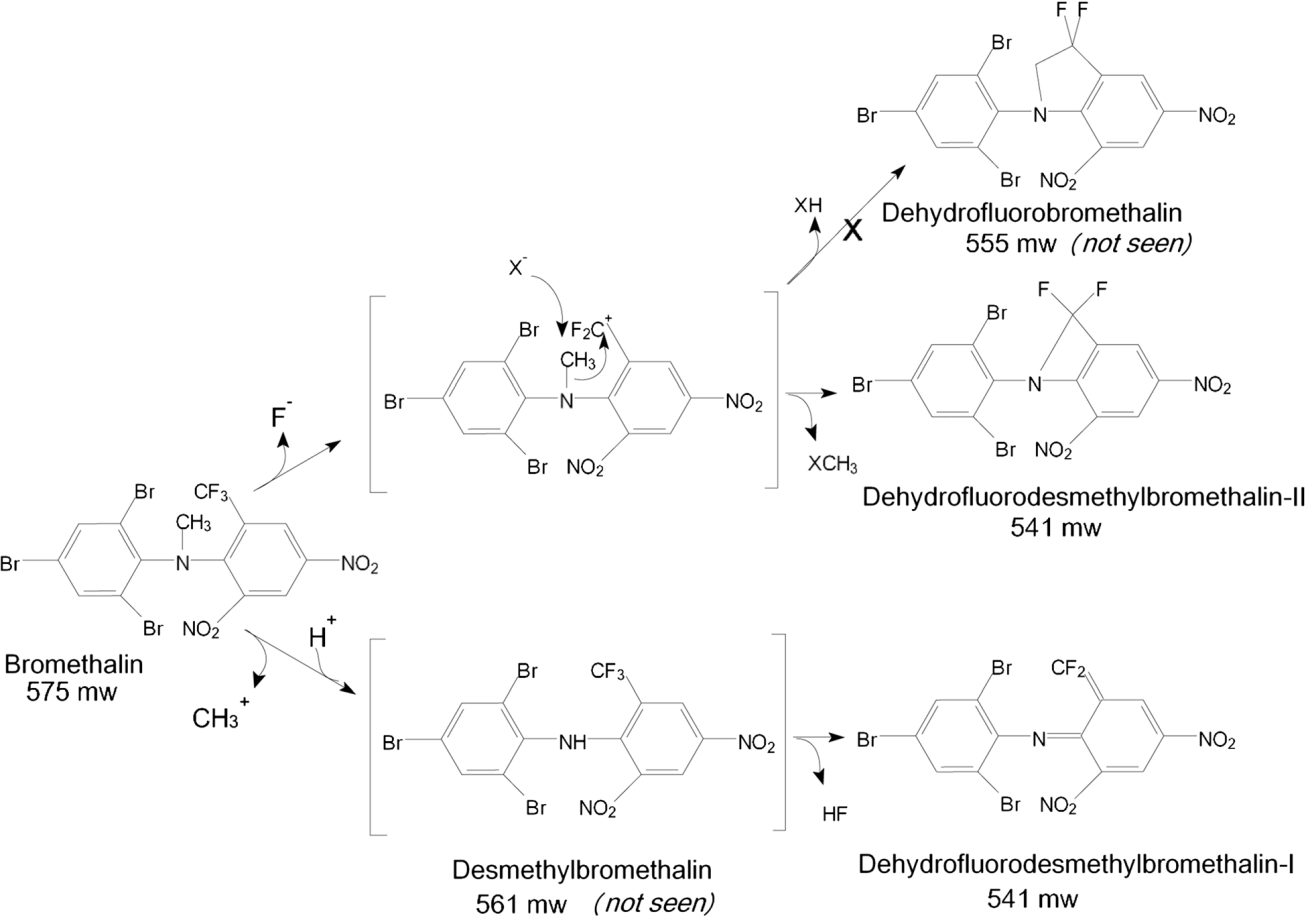
Likely origin of the two different 543.7 MW (nominal MW 541) bromethalin breakdown products as dehydrofluorodesmethylbromethalin structures of formula C_13_H_4_N_3_O_4_F_2_Br_3_. The compounds have been given common names of dehydrofluorodesmethylbromethalin-I and –II, which would be 6-(difluoromethylene)-2,4-dinitro-N-(2,4,6-tribromophenyl)cyclohexa-2,4-dien-1-imine and 8,8-difluoro-3,5-dinitro-(2,4,6-tribromophenyl)-7-azabicyclo[4.2.0]octa-1,3,5-triene, respectively. Dehydrofluorobromethalin (3,3-difluoro-5,7-dinitro-1-(2,4,6-tribromophenyl)indoline) was not seen and its absence is significant in light of the apparent lack of formation of a five member ring in deference to a more constrained four member ring

In the adipose and kidney samples provided for this case, a 543.7 MW breakdown product of molecular formula C_13_H_4_N_3_O_4_F_2_Br_3_ provided analytical evidence for bromethalin exposure (Fig. 3b,c). Figure 4 illustrates the likely origin of the two different 543.7 MW (nominal MW 541) bromethalin breakdown products. Both liver and brain were negative for bromethalin, desmethylbromethalin, and either breakdown product. Novel peaks were assigned by inference that compounds with tribrominated isotopic abundance patterns surrounding the molecular ion could only have arisen from bromethalin either in standards of bromethalin or in spiked liver samples (spectra not shown).

## Discussion

Bromethalin is a highly liposoluble compound that is rapidly absorbed after ingestion, undergoes enterohepatic circulation, and readily crosses the blood brain barrier. It is metabolized by P450 enzymes to its active metabolite desmethylbromethalin which uncouples oxidative phosphorylation in mitochondria, leading to a rapid decline in available ATP [8]. Subsequently, Na+/K+ pumps fail as their activity is dependent on energy derived from ATP. Increased intracellular sodium concentrations lead to cellular edema due to the passive influx of water through the plasma membrane, splitting of the myelin sheaths, and increased CSF pressure leading to neurologic signs and death [3, 8, 9]. In our case, the amount of bromethalin reported to be ingested by the owner was estimated to be low (0.8 mg/kg) and should have resulted in either no clinical signs or development of signs over several days. However, multiple factors support bromethalin’s role in this case including the nature of the clinical signs, persistent rigidity prior to death (suggesting loss of ATP function), as well as finding breakdown products of bromethalin in the animal’s tissue. One hypothesis for the sudden onset of severe clinical signs in this case is that the dog actually ingested either a larger amount of the mouse bait than was reported by the owner, or ingested additional bait days prior to the owner noticing. A likely alternative option is that the use of activated charcoal contributed to the acute development of the neurologic disorder. It has been reported that activated charcoal administration can lead to rare idiosyncratic hypernatremias in approximately 0.6 % of cases in humans (>155 mmol/L) [10, 11] and has been anecdotally reported as a rare occurrence in dogs [12].

Abrupt changes in natremic status can have devastating, even fatal neurologic consequences in a wide variety of animal species including humans, dogs, pigs, poultry and ruminants. Typical lesions include osmotic demyelination syndromes (e.g. abrupt increase in natremia to treat a hyponatremic status) or cortical laminar necrosis (e.g. salt intoxication/water deprivation/water intoxication that lead to an abrupt decrease in natremia after a hypernatremic status). Neurologic clinical signs and histologic lesions of the white matter in humans and dogs suffering from osmotic demyelination syndromes are somehow similar to those seen with bromethalin intoxication [3, 13]. With acute bromethalin intoxication changes are most commonly limited to myelin swelling (intra-myelinic edema) leading to increased intracranial pressure (e.g. 251 mm water in rats treated at 1 mg/kg compared to control value 68 mm water) [8] without evidence of myelin loss (as in our case, data not shown). Regarding osmotic demyelination syndromes, a myelin and oligodendrocyte loss is noted leading to central pontine myelinolysis and/or extrapontine myelinolysis; however clinical signs that are 2 to 6 days delayed after the swift elevation of sodium levels are often irreversible or only partially reversible [13, 14]. The central pontine myelinolysis lesions in dogs are virtually identical to those of human cases and the clinical course and manifestations are identical [15]. Why bromethalin is not inducing oligodendrocyte and myelin loss is not well understood. According to chronic and reversibility studies in rats, animals that survived the acute toxicity phase showed no evidence of myelin or oligodendrocyte loss [8].

The histologic changes induced by bromethalin are dose dependent [8] and white matter vacuolation can be minimal or even considered as an autolytic artifact. IHC for NeuN and GFAP can be utilized to characterize and confirm subtle changes. NeuN is a neuronal marker that shows strong reactivity in both surgical and post mortem samples with little variation in staining intensity regardless of routine fixation or autolysis [16]. Decreases in NeuN reactivity can help to identify a neuronal loss but is also indicative of metabolic stress/hypoxia [17]. In our case (Fig. 2) metabolic neuronal stress was likely secondary to hemorrhages and brain swelling. Among their many roles astrocytes play a major function in controlling the blood brain barrier efflux [18]. Increased intensity of GFAP immunoreactivity highlighted the marked increased number of astrocyte processes as well as increased size of cell bodies (astrocytosis), confirming a likely pre-mortem osmotic imbalance. Our case illustrates how quickly astrocytes can react, revealing marked evidence of astrocytosis in 2–3 hours (estimated time between the onset of clinical signs and death).

The meningeal hemorrhages noted in this case were perplexing as there is no report of meningeal hemorrhage with bromethalin intoxication. Meningeal hemorrhage can be seen with severe seizure activity, typically due to hyperthermia caused by increased muscle activity combined with a decrease in respiratory mechanism of cooling. Similarly, meningeal hemorrhage is often seen in dogs suffering from heatstroke [19]. This dog was reported to have severe muscle tremors and rigidity and laryngeal function and respiration may have been compromised potentially leading to hyperthermia. An alternative mechanism of hemorrhage might be attributed to the abrupt natremic status change. A case report of multiple infants suffering from salt intoxication reported multiple intracranial hemorrhages [20], and a report of fatal hypernatremia in a dog, also found intracranial hemorrhages associated with thrombosis [21].

Although the efficacy of retrieval of bromethalin residues in experimental studies is relatively high, in a diagnostic setting this is not typically the case due to the fact that bromethalin is rapidly photodegraded under incandescent and fluorescent light within a matter of hours [3], and because bromethalin does not seem very stable in tissues (Dr Buchweitz, personal communication). Neither bromethalin nor the active metabolite desmethylbromethalin were identified in any tissues submitted for toxicologic analysis. If bromethalin intoxication is suspected, it is important to remember to protect samples collected for toxicologic analysis from light to prevent or alleviate photodegradation. Identifying surrogate markers formed from the breakdown of bromethalin expands the repertoire of molecules available for analysis in the absence of the parent and active compound. In our case, a bromethalin product was recovered that is identical to one proposed by Pasquale-Styles et al. (2006) as arising from Chemical Ionization during mass spectrometric analysis [4]. This product was identified in the patient’s adipose tissue and kidney by GC-MS/MS, confirming exposure to bromethalin. Unfortunately, the novel dehydrofluorodesmethyl breakdown product was not recovered from the brain tissue; however, only a portion of brain was submitted for analysis which might have hindered the detectability.

Treatment recommendations for bromethalin toxicity include administration of activated charcoal (because of the enterohepatic circulation) and a lipid emulsion (as the compound is liposoluble) as well as hospitalization and monitoring if the dose ingested is expected to result in clinical signs. The use of osmotic diuretics such as mannitol has also been recommended at the onset of clinical signs, but is less commonly administered as its effects are transitory and cerebrospinal fluid pressures rise when the infusion is stopped [8]. Idiosyncratic hypernatremia due to activated charcoal is likely very rare. The use of multiple and low doses of activated charcoal have been recommended to lower the risk of idiosyncratic hypernatremia [1]. Veterinarians should also consider strongly recommending in-hospital monitoring after treatment for bromethalin intoxication, regardless of the dose ingested and especially if activated charcoal therapy is deemed necessary.

## Conclusions

This complicated case illustrates the difficulty in determining if the bromethalin alone, the activated charcoal alone, or the combination of both contributed to the development of brain lesions. IHC for GFAP and NeuN were helpful in characterizing subtle changes indicating ante-mortem tissue reaction. Application of GC-MS/MS to screen for additional surrogate markers such as the reported breakdown products described herein will be useful in future diagnostic work-ups in which bromethalin consumption has been observed or is suspected.

## Abbreviations

AMU: Atomic mass unit
DHFDMB: Dehydrofluorodesmethylbromethalin
GC/MS: Gas chromatography/mass spectrometry
GC-MS/MS: Gas chromatography tandem quadrupole mass spectrometry
GPC: Gel permeation column
GFAP: Glial fibrillary acidic protein
IHC: Immunohistochemistry
MRM: Multiple reaction monitoring
MW: Molecular weight
m/z: mass-to-charge ratio
NeuN: Neuron specific protein
RRT: Relative retention time
SIM: Selected ion monitoring
